# Can Serum GFAP and UCH-L1 Replace CT in Assessing Acute Ischemic Stroke Severity?

**DOI:** 10.3390/life15030495

**Published:** 2025-03-18

**Authors:** Ivan Kraljević, Maja Marinović Guić, Danijela Budimir Mršić, Krešimir Dolić, Krešimir Čaljkušić, Benjamin Benzon, Daniela Šupe Domić, Sanja Lovrić Kojundžić

**Affiliations:** 1 Clinical Department of Diagnostic and Interventional Radiology, University Hospital of Split, 21000 Split, Croatia; maja.marinovic.guic@gmail.com (M.M.G.); danijelabudimir@gmail.com (D.B.M.); kdolic79@gmail.com (K.D.); 2 Department of Diagnostic and Interventional Radiology, School of Medicine, University of Split, 21000 Split, Croatia; 3 Department of Health Studies, University of Split, 21000 Split, Croatia; daniela.supedomic@gmail.com; 4 Department of Neurology, University Hospital of Split, 21000 Split, Croatia; kresimir.caljkusic@gmail.com; 5 Department of Neurology, School of Medicine, University of Split, 21000 Split, Croatia; 6 Department of Anatomy, Histology and Embryology, School of Medicine, University of Split, 21000 Split, Croatia; benzon.benjamin@gmail.com; 7 Department of Anatomy, School of Medicine, University of Mostar, 88000 Mostar, Bosnia and Herzegovina; 8 Medical Laboratory Diagnostic Division, University Hospital of Split, 21000 Split, Croatia

**Keywords:** ischemic stroke, GFAP, UCH-L1, biomarker, computed tomography, perfusion, clot burden score

## Abstract

As acute ischemic stroke (AIS) is still a significant cause of morbidity globally, new methods of rapid diagnostics are continually being researched and improved. Still, the only definite way to diagnose AIS is radiological imaging. Lately, serum biomarkers glial fibrillary acidic protein (GFAP) and ubiquitin C-terminal hydrolase L1 (UCH-L1) have shown their usefulness in AIS as potential complementary tools in early recognition. We aimed to investigate if GFAP and UCH-L1 can correlate with comprehensive diagnostic information provided by computed tomography (CT) and several clinical parameters in AIS severity assessment and subsequently with clinical outcomes. Fifty-two patients with AIS and a potential for mechanical thrombectomy (MT) were included in our study. Thirty-seven patients underwent MT. Results showed no correlation of biomarkers with any analyzed CT parameter (thrombus length, volume, and density, clot burden score, collateral score, AIS core and penumbra volume, differences in perfusion between healthy and affected brain tissue). In addition, none of the clinical parameters, such as sex, symptom onset time, or the National Institutes of Health Stroke Scale, correlated with biomarkers. However, lower biomarker levels corresponded with a good clinical outcome, and higher levels to a poor outcome following hospital discharge, irrespective of the performed MT (*p* = 0.005 for GFAP, *p* = 0.001 for UCH-L1). In patients with successful MT, there were also differences between patients with a good clinical outcome compared with patients with a poor clinical outcome (*p* = 0.007 for GFAP, *p* = 0.004 for UCH-L1). In conclusion, these biomarkers cannot replace imaging modalities but can provide complementary diagnostic information in the setting of AIS.

## 1. Introduction

Stroke is still the second most common cause of death globally and is a significant contributor to disability [1]. It can be classified as either hemorrhagic or ischemic [2]. Hemorrhagic stroke (HS) develops following the rupture of a blood vessel, forcing blood to flow into the intracranial space, whereas acute ischemic stroke (AIS) occurs when there is a blockage within the blood vessel, restricting blood supply to the brain [1]. Although HS and AIS clinical presentations may overlap, they both have their own distinct treatment options that differ greatly—only AIS treatment has a limited time period from symptom onset 4.5 h for intravenous thrombolysis (IVT) or 6 to 24 h for mechanical thrombectomy (MT), depending on the occluded vessel [3,4,5]. Therefore, proper and timely diagnostics are essential.

Generally, the detection of stroke initially relies on clinical presentation and urgent neuroimaging by computed tomography (CT) or magnetic resonance imaging (MRI). However, potential serum biomarkers are increasingly being researched as complementary tools in early diagnostics. Two biomarkers recently researched in the setting of brain tissue damage are glial fibrillary acidic protein (GFAP) and ubiquitin C-terminal hydrolase L1 (UCH-L1). GFAP is a protein primarily expressed in astrocytes, and since it is not released under physiological conditions, its concentrations in the serum of healthy individuals are very low [6,7]. On the other hand, UCH-L1 is found in neurons and neuroendocrine cells, where it maintains self-repair mechanisms [8]. Based on the idea of measuring brain tissue damage, a combined serum biomarker of GFAP and UCH-L1 has been utilized in patients with mild traumatic brain injury prior to neuroimaging procedures [9]. Similarly, over the past few years, both biomarkers have also shown their usefulness in AIS patients, especially in differentiating AIS from healthy patients, or distinguishing AIS from HS [10,11]. Furthermore, recent studies showed that it was possible to differentiate between two different features of AIS based on GFAP and UCH-L1 serum levels—large vessel occlusion (LVO), which is eligible for MT, and small vessel occlusion (SVO) which is not [12,13]. Although no universal definition for LVO exists, it commonly includes the intracranial internal carotid artery (ICA), proximal segments of the middle cerebral artery (MCA); M1 and M2 segments, proximal anterior cerebral artery (ACA); A1 segment, or basilar artery (BA), as well as combinations of these vessels [14]. In contrast, SVO includes distal segments of all aforementioned vessels.

However, stroke severity can greatly differ between patients even if the same vessel is occluded and their clinical outcomes depend on a multitude of parameters [15]. Firstly, the starting time of symptoms can vary from “wake up” strokes (WUS), when it is impossible to determine the moment of occlusion, to the immediate reaction of the patient upon observing the start of his or her disability [16]. Secondly, epidemiological studies have shown that different age and sex is correlated to different results; older age and female sex are linked to a worse burden of stroke mortality and disability [17]. Moreover, some studies showed variability in GFAP biomarker levels between different age groups in healthy individuals and also variability in UCH-L1 levels between different sexes in a setting of mild brain trauma [18,19].

Furthermore, characteristics of the clot that occluded the vessel can provide useful clinical information and have been linked to different functional outcomes [20,21]. Presence of “hyperdense artery sign” (HAS) on unenhanced CT denoting visible clot in the occluded artery has been linked to worse outcomes [20]. Similarly, the extent of a clot in length or volume, subsequent clot burden score (CBS) determined on computed tomography angiography (CTA), and clot density, have also been used as predictors of clinical outcomes [21]. In addition, the development of leptomeningeal collaterals in the affected brain region, called collateral score (CS) can also influence successful recovery [22]. All of the mentioned parameters are then reflected in computed tomography perfusion (CTP) parameters, which can also predict the severity of AIS [23]. Occasionally, interventional radiologists use these criteria to assess a patient’s eligibility for MT.

In addition to the above-mentioned multiple clinical and radiological parameters, patients’ recovery also relies on the efficacy of IVT or MT, or a combination of both [24]. Unfortunately, not all MT are successful. They depend on the technique used by the interventional radiologist (aspiration, stent-retriever, or both), or on the number of attempts to extract the clot from the vessel (also called “number of passes”); as was shown the greater the number, the worse the outcome [25,26]. Finally, and most importantly, recovery relies on the amount of achieved reperfusion measured by the Modified Treatment in Cerebral Ischemia (mTICI) scale [27].

Although none of these parameters can be used on their own to precisely predict how a patient will recover after AIS, they can offer valuable insight into what to expect from a clinical standpoint and help interventional radiologists decide whether to perform MT or not. While serum biomarkers, GFAP and UCH-L1, at the moment lack the wide array of information that diagnostic imaging can provide, emerging new studies demonstrate their potential to be complementary prehospital screening tools in AIS. A recent study by Florijn et al. showed that GFAP can differentiate stroke subtypes, and in combination with UCH-L1 can improve test sensitivity and specificity [28]. Furthermore, a study by Herrmann et al. correlated GFAP and another neuronal biomarker S-100B with stroke lesion volumes in scans of unenhanced CT calculating volumes of well-defined infarct areas [29]. Another study by Pujol-Calderón et al. also correlated several neuronal biomarkers, including GFAP, with stroke volumes calculated with an unenhanced CT, and also with clinical outcomes after 3 months with positive results [30]. However, no study investigated correlation of extensive CT parameters available at symptom presentation with these biomarkers. Also, volumes measured with unenhanced CT can provide no helpful information in the moment of treatment decision making unlike volumes measured by perfusion. Lastly, few studies regarding neuronal biomarkers in the setting of AIS test UCH-L1 as extensively as others. Therefore, our aim was to deepen the knowledge of these serum biomarkers in AIS patients and to provide new data for potential clinical use by investigation of unknown relationship of GFAP and UCH-L1 levels with comprehensive imaging and clinical parameters.

## 2. Materials and Methods

### 2.1. Study Design and Participants

When patients presented with suspected AIS (presence of hemiparesis at hospital admission (i.e., unilateral paresis of arm and leg) and/or presence of at least one clinical sign of hemispheric involvement, such as aphasia, neglect, homonymous hemianopia, gaze deviation to the contralateral side of the hemiparesis, or reduced level of consciousness) to the Emergency Department of the University Hospital of Split, Croatia, they underwent standard medical evaluation, as well as assessment using the National Institutes of Health Stroke Scale (NIHSS) by the attending neurologist [31]. Blood samples were taken at admission for standard laboratory workup and an additional sample for GFAP and UCH-L1. If the symptoms started within the last 6 h, or it was a WUS, rapid neuroimaging protocol for AIS was ordered—unenhanced brain CT, CTA, and CTP. Experienced radiologists and interventional radiologists confirmed or rejected the diagnosis.

Based on clinical and imaging data, inclusion criteria were as follows: (a) admission within 6 h of symptom onset, or WUS, (b) radiologically verified AIS caused by LVO.

Exclusion criteria were as follows: (a) other etiologies causing AIS symptoms (transient ischemic attack, head trauma, HS, intracranial tumors, and severe brain edema), (b) demarcated lesions on CT corresponding to previous stroke, (c) any history of diagnosis of brain tumor at any time in their medical history.

In the period from March 2023 to November 2023, 52 patients met our inclusion criteria. After study inclusion, the following variables were collected from the hospital information system: patient demographics and history (age, sex, risk factors, and comorbidities), clinical data (NIHSS on admission, mTICI score), radiological data (HAS, clot density, length, and volume, CBS, CS, and CTP characteristics), general laboratory findings, patients’ clinical recovery at discharge quantified using the modified Ranking scale (mRS) (scores 0–2 were considered a good clinical outcome and scores 3–6 were considered poor clinical outcome, with 6 including patient death) [32].

### 2.2. Blood Sample Collection and Processing

As part of the standard workup upon hospital admission, blood samples were obtained, and each patient also had extra samples taken for GFAP and UCH-L1. Blood tubes were taken to the hospital’s laboratory and centrifuged at 1500× *g* for 10 min within 60 min of blood collection. The serum was then separated into 0.5 mL aliquots and stored at −80 °C. Following the identification of the patient with AIS caused by LVO, blood samples were recovered from the storage. We did not analyze blood samples from patients who did not fit our inclusion criteria. Serum samples were handled by board-certified laboratory technicians who were blinded to clinical data. The commercial Abbottkit (04W1720), Abbott, Sligo, Ireland, was used to measure levels of UCH-L1 and GFAP in the serum. All measurements were conducted in full calibration mode. According to the definitions of CLSI guidelines EP34, 1. edition, the ranges of measurable values within which the results can be reported are based on representative data for the limit of quantification and limit of detection and are, for GFAP 6.1–42,000 pg/mL and for UCH-L126.3–25,000 pg/mL [33].

### 2.3. Imaging Data and Analysis

Using a 128-slice CT Siemens Somatom, Erlangen, Germany, all patients underwent a standardized neuroimaging protocol for AIS, analyzed using the Syngovia software (version VB60A, Hofix05), Siemens Healthineers, Erlangen, Germany. Two experienced radiologists, including an interventional radiologist, analyzed unenhanced brain CT, CTA, and CTP for signs of AIS (hypodense area on unenhanced CT, positive HAS, vessel occlusion, lowered brain perfusion with a core/penumbra mismatch ratio denoting salvageable brain tissue in the penumbra area), and if there were indications for MT. Afterwards, additional information was obtained from available imaging of LVO patients whose serum samples were analyzed. We defined LVO as an occlusion of ICA, M1, M2, A1, BA, or any combination of those vessels, such as T-occlusion (distal ICA, M1, and A1) or tandem occlusion (distal ICA and M1). Readers were blinded to clinical information.

**Figure 1:**
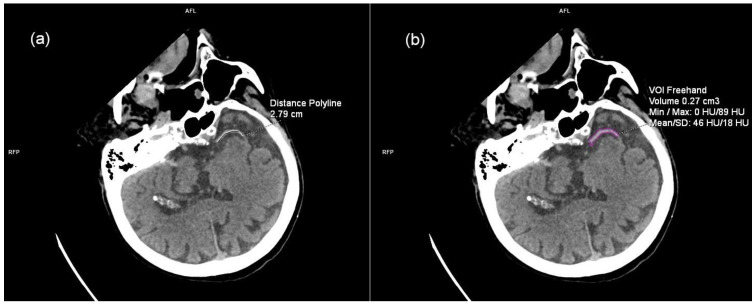
Thrombus characteristics measured on unenhanced brain CT after multiplanar positioning to show the full extent of the hyperdensity. (**a**) Thrombus length measured by distance polyline. (**b**) Thrombus volume and density measured by free-hand VOI. VOI = volume of interest; HU = Hounsfield units; SD = standard deviation.

On unenhanced CT, we looked for the presence of HAS in the occluded vessel. If the noted hyperdensity correlated with the lack of contrast opacification in the same vessel on CTA, it was considered as positive HAS. In those patients with positive HAS, it was possible to measure the length of the clot. After multiplanar positioning to show the full extent of the hyperdensity, length was measured by Distance Polyline (Figure 1a). Similarly, free-hand volume of interest (VOI) was used to measure clot volume. Hyperdensity inside the vessel was marked on each slice and the volume was computer generated based on marked areas (Figure 1b). When measuring VOI, the computer also calculates clot density.

On CTA, we analyzed CBS and CS for anterior circulation, according to previously reported measuring guidelines [21,22]. When calculating CBS, each ICA with its two main branches, MCA and ACA, was given 10 points for the presence of contrast opacification. Two points each were subtracted for the absence of contrast agent in any part of the proximal M1 segment, distal M1 segment or supraclinoid ICA and one point each for M2 branches, A1 segment and infraclinoid ICA. A score of 10 indicates absence of a visible occlusion on CTA, while a score of 0 indicates occlusion of all major anterior circulation arteries. When grading CS, a scale of 0–3 was used; a score of 0 indicated absent collateral supply to the occluded MCA territory, a score of 1 indicated collateral supply filling 0–50%, a score of 2 for collateral supply filling 50–100%, and a score of 3 was given for 100% collateral supply of the occluded MCA territory.

**Figure 2:**
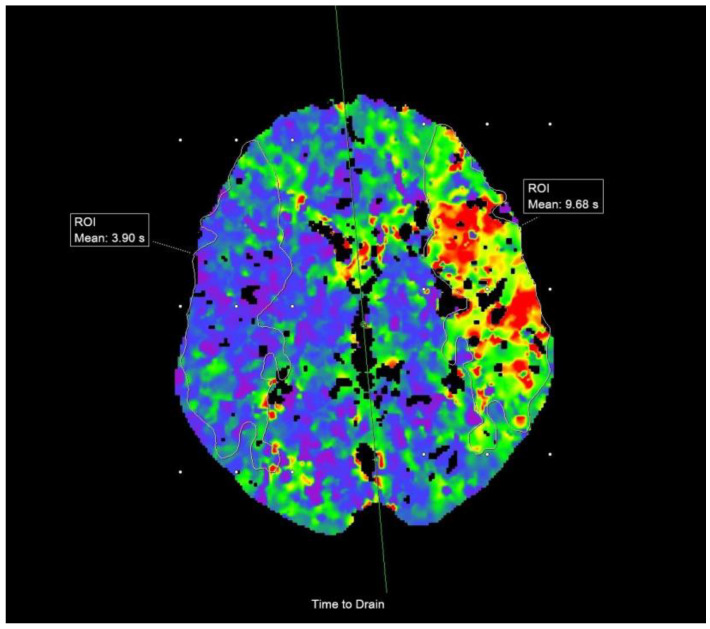
Time-to-drain on CTP showing increased values in the affected left hemisphere and normal values in the symmetrical area in the healthy right hemisphere, measured by free-hand ROI. ROI = region of Interest.

Finally, from CTP imaging, we collected data on ischemic lesion core volume and penumbra volume, which were computer generated. We also analyzed cerebral blood flow (CBF), cerebral blood volume (CBV), mean transit time (MTT), and time-to-drain (TTD). We used free-hand region of interest (ROI) to measure each parameter in the lesion area, with computer generating symmetrical area on the healthy, contralateral side (Figure 2). Afterwards we calculated the difference in said parameters between the affected and the healthy side.

### 2.4. Statistical Analysis

Continuous variables are presented as median and interquartile range, discrete variables are presented as percentages. *t* test for unpaired samples with or without Welch correction was used to test for differences in means of continuous data, if needed continuous variables were log transformed. Correlations were quantified with Pearson’s correlation coefficient for continuous variables and with Spearman’s correlation coefficient for rank variables, i.e., scores. *p* values were used as measure of evidence, along with the correlation coefficients.

## 3. Results

This study included a total of 52 participants. The median age was 78, with the youngest patient being 52 years old, and the oldest 93 years old. Other baseline characteristics are shown in Table 1 and laboratory parameters in Table 2.

Serum concentrations of GFAP and UCH-L1 were analyzed for any correlation with several imaging (CT, CTA, and CTP) parameters; thrombus length, volume, and density, CBS, CS, AIS core and penumbra volume, difference between healthy and affected CBF, CBV, MTT, and TTD. None of these parameters showed correlation with biomarker serum levels (Table 3).

We compared the biomarker levels of patients with a good clinical outcome (median: 129.7 pg/mL for GFAP, 288.2 pg/mL for UCH-L1) to those with a poor outcome (median: 493 pg/mL for GFAP, 591.1 pg/mL for UCH-L1) by analyzing the mRS of all patients following hospital discharge, irrespective of the performed MT and found differences for both GFAP (*p* = 0.005) and UCH-L1 (*p* = 0.001). MT was performed on 37 patients, while 15 were deemed unfit for the procedure by the interventional radiologist with causes shown in Table 4.

**Figure 3:**
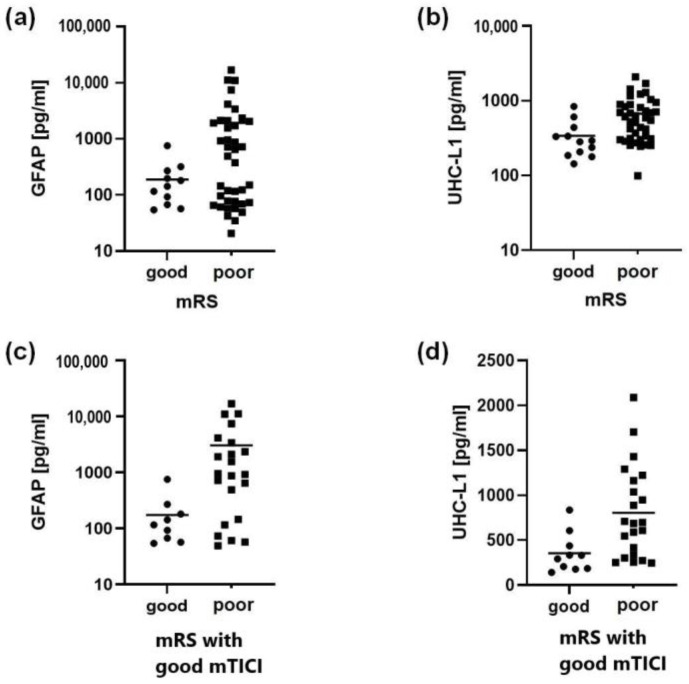
Relationship between good and poor mRS outcomes for both GFAP and UCH-L1. Data are presented as medians—circles for good mRS group and squares for poor mRS group. (**a**,**b**) All patients were analyzed, regardless of eventual MT. (**c**,**d**) Only patients with successful recanalization were analyzed. In all cases, patients with poor clinical outcomes had higher levels of serum biomarkers. GFAP = glial fibrillary acidic protein; UCH-L1 = ubiquitin C-terminal hydrolase-L1; mRS = modified Rankin scale; mTICI = Modified Treatment in Cerebral Ischemia.

We then analyzed only patients with successful recanalization (mTICI > 2a) and showed that there were also measurable differences in biomarker levels for both GFAP (*p* = 0.007) and UCH-L1 (*p* = 0.004) between patients with a good clinical outcome (median: 104.6 for GFAP, 312.8 for UCH-L1) opposed to patients with a poor outcome (median: 943.9 for GFAP, 692.3 for UCH-L1) (Table 5 and Figure 3).

Additionally, we compared all previously analyzed imaging parameters (as stated in Table 3) with biomarker levels in patients grouped by their clinical outcomes based on mRS scores. In patients with good outcomes, only thrombus length and thrombus volume showed correlation with GFAP serum concentration (*p* = 0.011, *p* = 0.042, respectively), while UCH-L1 showed correlation to ischemic core volume (*p* = 0.007). No other parameter in either group showed correlation with biomarker serum levels (Table 6).

Finally, the several clinical parameters that could potentially cause differences in serum biomarkers levels were also analyzed. When taking sex into account, no significant difference between levels of both biomarkers was found (*p* = 0.871 for GFAP, *p* = 0.345 for UCH-L1). Similarly, in a group of patients with WUS, which did not have a specific onset time, and in a group of patients that presented and were treated within 6 h after AIS occurrence, no significant difference was confirmed (*p* = 0.708 for GFAP, *p* = 0.08 for UCH-L1). Lastly, no serum level differences between patients that presented with favorable NIHSS score (0–15) and those with unfavorable (16–42) were found (*p* = 0.102 for GFAP, *p* = 0.194 for UCH-L1) (Table 7).

## 4. Discussion

Our study showed that patients with poor clinical outcomes had higher GFAP and UCH-L1 serum levels compared to patients with good clinical outcomes, regardless of if MT was performed or not. Also, none of the abundant imaging parameters used for AIS assessment (thrombus length, volume and density, CBS, CS, AIS core and penumbra volume, difference between healthy and affected CBF, CBV, MTT and TTD), as well as clinical parameters such as sex, WUS or NIHSS, showed correlation with biomarker serum levels. When analyzing the same imaging parameters in patients grouped by clinical outcomes, those with a good recovery showed correlation in only few variables with either GFAP or UCH-L1. On the other hand, those with a poor recovery showed no correlation.

Biomarker UCH-L1 is a brain-specific protein that resides in neurons and accounts for up to 5% of all soluble brain protein [34]. It is essential for maintaining axonal integrity and plays a role in eliminating excess, oxidized, or misfolded proteins in both healthy and neuropathological circumstances [35]. With insurmountable stress upon the cell, such as ischemia, UCH-L1 becomes overwhelmed in its task, which leads to subsequent neuronal death and its release in cerebrospinal fluid and in serum [36,37]. Similarly, GFAP is a brain-specific protein, but it resides in astrocytes [7]. Astrocytes are a subtype of glial cells that comprise the predominance of cells in the human central nervous system. Their function is to maintain brain homeostasis by tasks such as stabilizing and regulating the blood–brain barrier [38]. In many neurological disorders, such as trauma and ischemia, they react with astrogliosis, which is a reparative endeavor to salvage damaged brain tissue by proliferating and sequestering the lesion, and in severe cases causing a glial scar, thus stopping further injury [39]. In this process, reactive astrocytes show altered expression of many genes, mainly upregulation of GFAP, which is later also detectable in serum [40].

Taking into account these biological aspects of GFAP and UCH-L1 (release in serum proportional to amount of damaged brain tissue), it is unexpected that they did not show connection with infarcted core volume. Research by d’Esterre et al. provides one way of explaining this, as they showed that CTP images only reflect the tissue’s “perfusion-state” and actually provide no direct insight of the amount of tissue that has sustained irreparable damage [41]. In addition, patients that undergo rapid neuroimaging in a hyperacute state (within 1 h of symptom onset), require a more restrictive CBF threshold for adequate CTP information to determine the ischemic core [42]. However, in the setting of an emergency, it can be detrimental to spend precious time adjusting thresholds for each patient, and sometimes we do not even know the exact starting time of the symptoms. Therefore, CTP can be misleading in acute patients, and the use of MRI could provide more accurate information if needed [43]. On the other hand, we did not presume that the penumbra volume would show correlation with biomarkers, as it is a zone of hypoperfusion with not yet realized cell death. The same can be said for all other perfusion parameters, which are a reflection of currently affected tissue, be it a large volume or a small one. This was consistent with our findings.

As mentioned above, sometimes we do not know the exact starting time of symptoms, such as in WUS patients. That can pose a problem, as some studies have shown that the release of both GFAP and UCH-L1 is time dependent [44,45]. Our study included 10 such patients who did not differ in biomarker serum levels from patients with a known symptom onset time. An explanation can be found in the results of a study by Cheng et al., which was a part of a large WAKE-UP trial [46]. Using MRI, they investigated nocturnal stroke onset times by taking into account when patients were last seen well and when first symptoms were recognized and concluded that AIS occurs during the early morning hours, much closer to the first recognition of symptoms. Thus, if WUS happens closer to regular symptom onset time, they could be considered “normal” AIS, which does not affect the time dependable change in the release of biomarkers, which would be in accordance with our results.

Additionally, no thrombus characteristics demonstrated an effect on biomarker serum levels. Several studies showed that thrombus length, volume, and density independently can coincide with better or worse clinical outcomes, yet our study showed that the affected brain tissue remained the same regardless of these factors [47,48]. Once a vessel is blocked, it causes hypoperfusion of neurons distally and everything proximal to the occlusion site is irrelevant, as it cannot additionally lower the blood flow in the same area. Even though a thrombus might be large in its size, or hard as suggested by its density, it will only be more difficult for IVT or MT treatment—the larger or harder the thrombus it will be harder to extract and dissolve [47,48]. Correlation of CBS with biomarker levels is explainable in the same manner. Although the CBS depends on the thrombus extent within the vessel, only the primarily proximal occluded site is important with an “all or nothing” effect and with subsequent distal propagation of the thrombus [49].

On the other hand, CS is a parameter of the brain’s potential for tissue salvation. As such, a lack of correlation with biomarker serum levels is not unexpected. Even though there is a theoretical possibility that well-developed leptomeningeal collateral network could manage to reperfuse stroke core, we did not find this to be the case. It can only delay the progression of salvageable penumbra to irreversible tissue death and consequently affect clinical outcomes [22].

Furthermore, our results showed that the larger the GFAP and UCH-L1 levels, the worse the clinical outcomes are. As these biomarkers reflect the amount of already damaged tissue, these results are expected and, for GFAP are in accordance with a recent review by Anogianakis et al., while there are no comparable results for UCH-L1 [50]. Whether the patient received any therapy or not, had little effect on the results, as the injury had already happened before any intervention. On that note, we analyzed all imaging parameters in groups based on clinical outcomes in order to test the possibility of prognosticating stroke outcomes. We found that GFAP correlated with thrombus length and volume, and UCH-L1 with ischemic core volume in the group with a good clinical result, while no parameter was correlated with any biomarker in the group with poor results. We could not find a satisfactorily biological or pathophysiological explanation for only these few variables, and it is more likely that they reflect a poor statistical power of the group.

Moreover, we studied NIHSS score which presents patient’s clinical state at admission and is a good predictor of stroke severity and clinical outcome [51]. However, our results did not correlate this with GFAP and UCH-L1 levels. Several studies explored the same parameters and reported conflicting reports; Surjawan et al. and Yao et al. displayed a positive correlation between GFAP serum levels and NIHSS score, while Yigit et al. showed no correlation between UCH-L1 and NIHSS score, and another study showed no correlation between both GFAP and UCH-L1 with NIHSS score [12,52,53,54]. There are differences between all of these studies; patient’s blood samples were not acquired in the same time period and groups were inconsistently divided by NIHSS score. Nevertheless, this metric is ultimately predicated on the patient’s clinical status at the outset of symptoms, when neuronal damage may not have yet occurred. At the time of our testing, hypoperfusion of salvageable brain tissue in the penumbra zone may result in severe symptoms without increasing biomarker serum levels.

Finally, our results demonstrated no differences between male and female patients, which is not in alignment with the study by Papa et al. They showed that patient’s sex could affect biomarker levels; male patients had greater levels of UCH-L1 than female, while GFAP did not show comparable results [19]. These differences might be exhibited in a physiological setting, while AIS elevates biomarkers to such levels where groups are indiscernible.

One limitation of our study is the lack of information for mRS after 90 days. We analyzed mRS at discharge, while mRS after 90 days was available only for nine patients, disregarding 12 patients who passed away during the initial hospital stay. The other 31 were not available due to either never coming for a control follow-up or due to seeking further treatment in another medical institution, which is not uncommon, as some of our patients are tourists here during the summer season who prefer to receive treatment in their own country. Another limitation is the relatively small number of included patients. A larger number would allow us to divide all the patients who received MT into smaller groups based on MT technique and the “number of passes” needed to achieve reperfusion. Finally, cooperation with other stroke centers would provide us with a larger potential patient population and an insight into variations in decision making or patient care, as this research is limited to a single center experience.

Despite the mentioned limitations, the main advantage of this study is a further deepened insight into the usefulness of both biomarkers GFAP and UCH-L1, since increased values indicate a clinically worse prognosis. Furthermore, the study demonstrated limited reliability of CTP when used within 6 h of symptom onset and found no correlation with multiple CT and CTA parameters. This highlights the need for a multidisciplinary approach to AIS given the current challenges in rapid diagnostics and the need for interventional radiologists to exercise greater caution and scrutiny when deciding whether to perform MT or not.

## 5. Conclusions

In conclusion, higher elevated levels of serum biomarkers GFAP and UCH-L1 showed correlation with worse clinical outcomes at hospital discharge for AIS patients, regardless of whether they received endovascular treatment or not. On the other hand, biomarkers did not correlate with comprehensive imaging parameters (thrombus length, volume, and density, CBS, CS, AIS core, and penumbra volume, difference between healthy and affected CBF, CBV, MTT, and TTD) and some clinical parameters (sex, WUS, or NIHSS) used in diagnostics and decision-making regarding MT. This demonstrates that these biomarkers still cannot replace imaging modalities but can provide complementary diagnostic information in the setting of AIS.

## Figures and Tables

**Table 1:** Baseline characteristics of the study population. Data are given as a number and percentage.

	*n* = 52
Women (*n*, %)	34 (65.4%)
Arterial hypertension (*n*, %)	35 (67.3%)
Diabetes mellitus (*n*, %)	9 (17.3%)
Atrial fibrillation (*n*, %)	25 (48.1%)
Hyperlipidemia (*n*, %)	11 (21.2%)
Known malignancy (*n*, %)	6 (11.5%)

**Table 2:** General laboratory parameters of the study population collected at admission. Data are given as median with interquartile range.

	Median (IQR), *n* = 52
Leukocytes (×10^9^/L)	8.4 (6.9–11.68)
Erythrocytes (×10^12^/L)	4.6 (4.05–4.97)
Hemoglobin (g/L)	137 (127.3–149.8)
Hematocrit (L/L)	0.4 (0.38–0.44)
Thrombocytes (×10^9^/L)	211 (160.8–273.5)
PT (s)	1.05 (0.85–1.16)
Glucose (mmol/L)	7.15 (6.33–8.68)
Urea (mmol/L)	7 (5.43–9.43)
Creatinine (µmol/L)	83 (72–101.5)
AST (U/L)	22 (18–27)
ALT (U/L)	16 (13–27.75)
GGT (U/L)	23.5 (16–39)
LDH (U/L)	180.5 (34.25–230.5)
CRP (mg/L)	3.45 (1.53–13.38)
Na^+^ (mmol/L)	141 (140–142.8)
K^+^ (mmol/L)	4 (3.43–4.28)
Cl^−^ (mmol/L)	103 (101–106)

IQR = interquartile range; PT = prothrombin time; AST = aspartate transaminase; ALT = alanine transaminase; GGT = gamma-glutamyltransferase; LDH = lactate dehydrogenase; CRP = C-reactive protein; Na^+^ = sodium; K^+^ = potassium; Cl^−^ = chloride.

**Table 3:** Correlation of several CT parameters in AIS severity assessment with GFAP and UCH-L1.

	CT Parameter	*p* Value	*R* Value
GFAP (*n* = 51)	thrombus length *	0.586	0.077
	thrombus volume *	0.834	0.029
	thrombus density *	0.428	0.112
	CBS ^#^	0.157	−0.199
	CS ^#^	0.489	−0.098
	ischemic core volume *	0.177	0.190
	ischemic penumbra volume *	0.428	0.112
	CBF *	0.789	−0.038
	CBV *	0.536	0.088
	MTT *	0.310	−0.144
	TTD *	0.742	−0.047
UCH-L1 (*n* = 52)	thrombus length *	0.999	<0.001
	thrombus volume *	0.866	−0.024
	thrombus density *	0.710	−0.053
	CBS ^#^	0.291	−0.149
	CS ^#^	0.318	−0.141
	ischemic core volume *	0.679	0.059
	ischemic penumbra volume *	0.708	−0.053
	CBF *	0.296	−0.148
	CBV *	0.907	−0.017
	MTT *	0.130	−0.213
	TTD *	0.341	−0.135

* Pearson’s correlation; ^#^ Spearman’s correlation; CT—computed tomography; GFAP = glial fibrillary acidic protein; UCH-L1 = ubiquitin C-terminal hydrolase-L1; CBS = clot burden score; CS = collateral score; CBF = cerebral blood flow; CBV= cerebral blood volume; MTT = mean transit time; TTD = time-to-drain. Note: One patient did not have data for GFAP serum level due to technical reasons; therefore, their score was N/A for statistical analysis.

**Table 4:** Number of patients who did not receive MT with corresponding causes.

No. of Patients Unfit for MT	Cause
5	unfavorable core/penumbra ratio
3	extremely tortuous vascular anatomy for catheter manipulation
2	extensive carotid thrombosis
1	complete recanalization of the occluded vessel after only IVT
1	too low NIHSS (3)
1	substantial calcifications of the arteries at the puncture site
1	shortage of material needed for the procedure
1	no data

MT = mechanical thrombectomy; IVT = intravenous thrombolysis; NIHSS = National Institutes of Health Stroke Scale.

**Table 5:** GFAP and UCH-L1 serum levels in all LVO patients with good and poor clinical recovery, and patients after successful recanalization. Data are given as median and interquartile range.

		GFAP (pg/mL) (Median, IQR)	*p* Value	UCH-L1 (pg/mL) (Median, IQR)	*p* Value
all patients	good mRS (*n* = 12)	129.7 (59.48–252.4)	0.005 *	288.2 (191–413.5)	0.001 *
	poor mRS (*n* = 39)	493 (73.1–2046)		591.1 (309.7–889.4)	
successful recanalization	good mRS (*n* = 10)	104.6 (56.2–203.8)	0.007 *	312.8 (183.8–481.6)	0.004 ^#^
	poor mRS (*n* = 22)	943.9 (137.8–3599)		692.3 (328.4–1179)	

* Unpaired *t*-test with Welch’s correction; ^#^ unpaired *t*-test; GFAP = glial fibrillary acidic protein; UCH-L1 = ubiquitin C-terminal hydrolase-L1; IQR = interquartile range; mRS = modified Rankin scale.

**Table 6:** Correlation of several CT parameters with GFAP and UCH-L1 in patient groups with different clinical outcomes.

		GFAP		UCH-L1	
	CT Parameter	*p* Value	*R* Value	*p* Value	*R* Value
good mRS (*n* = 12)	thrombus length *	0.011	0.705	0.992	0.003
	thrombus volume *	0.042	0.593	0.899	−0.041
	thrombus density *	0.636	0.153	0.955	−0.018
	CBS ^#^	0.372	−0.282	0.899	−0.042
	CS ^#^	0.713	−0.122	0.766	−0.099
	ischemic core volume *	0.773	−0.093	0.007	0.727
	ischemic penumbra volume *	0.347	−0.298	0.849	−0.061
	CBF *	0.581	−0.178	0.812	−0.078
	CBV *	0.573	0.181	0.979	−0.008
	MTT *	0.271	−0.346	0.599	−0.169
	TTD *	0.978	0.009	0.486	0.223
poor mRS (*n* = 39)	thrombus length *	0.762	0.050	0.793	−0.044
	thrombus volume *	0.979	−0.004	0.654	−0.074
	thrombus density *	0.415	0.134	0.724	−0.059
	CBS ^#^	0.158	−0.230	0.304	0.169
	CS ^#^	0.818	−0.038	0.617	−0.083
	ischemic core volume *	0.349	0.154	0.782	−0.046
	ischemic penumbra volume *	0.467	0.119	0.689	−0.066
	CBF *	0.826	−0.036	0.328	−0.161
	CBV *	0.812	0.039	0.512	−0.108
	MTT *	0.699	−0.064	0.573	−0.093
	TTD *	0.973	0.006	0.569	−0.094

* Pearson’s correlation; ^#^ Spearman’s correlation; GFAP = glial fibrillary acidic protein; UCH-L1 = ubiquitin C-terminal hydrolase-L1; CT—computed tomography; mRS = modified Rankin scale; CBS = clot burden score; CS = collateral score; CBF = cerebral blood flow; CBV = cerebral blood volume; MTT = mean transit time; TTD = time-to-drain.

**Table 7:** Correlation of several clinical parameters (sex, symptom onset time, NIHSS) with GFAP and UCH-L1 serum levels. Data are given as median and interquartile range.

		GFAP (pg/mL) (Median, IQR)	*p* Value	UCH-L1 (pg/mL) (Median, IQR)	*p* Value
sex	male (*n* = 34)	147.7 (71.1–1602)	0.871 *	569.1 (296.1−849.9)	0.345 *
	female (*n* = 18)	250.7 (61.1−1064)		395.3 (283.5−628.1)	
symptom onset	WUS (*n* = 10)	132.3 (72.9−810.8)	0.708 ^#^	709.1 (253.8−1276)	0.080 *
	within 6 h (*n* = 42)	189.4 (66.9−1602)		429.7 (291.7−701.2)	
NIHSS	favorable (*n* = 14)	207.9 (92.2−2175)	0.102 *	551.6 (290.6−977.2)	0.194 *
	unfavorable (*n* = 19)	92.8 (57.4−319.8)		354.3 (273.4−698.5)	

* Unpaired *t*-test; ^#^ unpaired *t*-test with Welch’s correction; GFAP = glial fibrillary acidic protein; UCH-L1 = ubiquitin C-terminal hydrolase-L1; IQR = interquartile range; WUS = “wake up” stroke; NIHSS = National Institutes of Health Stroke Scale. Note: Some patients did not have a complete medical history; therefore, their NIHSS scores were N/A for statistical analysis.

## Data Availability

Data are contained within the article. The datasets are available upon reasonable request to the corresponding author.

## Abbreviations

The following abbreviations are used in this manuscript:

HS	Hemorrhagic Stroke
AIS	Acute Ischemic Stroke
IVT	Intravenous Thrombolysis
MT	Mechanical Thrombectomy
CT	Computed Tomography
MRI	Magnetic Resonance Imaging
GFAP	Glial Fibrillary Acidic Protein
UCH-L1	Ubiquitin C-terminal Hydrolase L1
LVO	Large Vessel Occlusion
SVO	Small Vessel Occlusion
ICA	Internal Carotid Artery
MCA	Middle Cerebral Artery
ACA	Anterior Cerebral Artery
BA	Basilar Artery
WUS	Wake up Stroke
HAS	Hyperdense Artery Sign
CBS	Clot Burden Score
CTA	Computed Tomography Angiography
CS	Collateral Score
CTP	Computed Tomography Perfusion
mTICI	Modified Treatment in Cerebral Ischemia
NIHSS	National Institutes of Health Stroke Scale
mRS	Modified Ranking Scale
VOI	Volume of Interest
CBF	Cerebral Blood Flow
CBV	Cerebral Blood Volume
MTT	Mean Transit Time
TTD	Time-to-Drain
ROI	Region of Interest
